# Development and Psychometric Evaluation of the Brief Adolescent Gambling Screen (BAGS)

**DOI:** 10.3389/fpsyg.2017.02204

**Published:** 2017-12-19

**Authors:** Randy Stinchfield, Harold Wynne, Jamie Wiebe, Joel Tremblay

**Affiliations:** ^1^Department of Psychiatry, University of Minnesota Medical School, Minneapolis, MN, United States; ^2^Department of Psychology, University of Windsor, Windsor, ON, Canada; ^3^Responsible Gambling Council, Toronto, ON, Canada; ^4^Department of Psychoeducation, Université du Québec à Trois-Rivières, Quebec, QC, Canada

**Keywords:** adolescent problem gambling, youth problem gambling, brief screen, classification accuracy, psychometric evaluation of brief screen

## Abstract

The purpose of this study was to develop and evaluate the initial reliability, validity and classification accuracy of a new brief screen for adolescent problem gambling. The three-item Brief Adolescent Gambling Screen (BAGS) was derived from the nine-item Gambling Problem Severity Subscale (GPSS) of the Canadian Adolescent Gambling Inventory (CAGI) using a secondary analysis of existing CAGI data. The sample of 105 adolescents included 49 females and 56 males from Canada who completed the CAGI, a self-administered measure of DSM-IV diagnostic criteria for Pathological Gambling, and a clinician-administered diagnostic interview including the DSM-IV diagnostic criteria for Pathological Gambling (both of which were adapted to yield DSM-5 Gambling Disorder diagnosis). A stepwise multivariate discriminant function analysis selected three GPSS items as the best predictors of a diagnosis of Gambling Disorder. The BAGS demonstrated satisfactory estimates of reliability, validity and classification accuracy and was equivalent to the nine-item GPSS of the CAGI and the BAGS was more accurate than the SOGS-RA. The BAGS estimates of classification accuracy include *hit rate* = 0.95, *sensitivity* = 0.88, *specificity* = 0.98, *false positive rate* = 0.02, and *false negative rate* = 0.12. Since these classification estimates are preliminary, derived from a relatively small sample size, and based upon the same sample from which the items were selected, it will be important to cross-validate the BAGS with larger and more diverse samples. The BAGS should be evaluated for use as a screening tool in both clinical and school settings as well as epidemiological surveys.

## Introduction

A number of brief screens have been developed for adult problem gambling, some as brief as one or two questions, but there are no brief screens for adolescent problem gambling (Stinchfield, 2010, 2014). There are four main assessment tools for adolescent problem gambling and they are the South Oaks Gambling Scale—Revised for Adolescents (SOGS-RA) (Winters et al., 1993, 1995); DSM-IV revised for Juveniles or DSM-IV-J (Fisher, 1992, 2000); Massachusetts Gambling Screen (MAGS) (Shaffer et al., 1994); and the Canadian Adolescent Gambling Inventory (CAGI) (Tremblay et al., 2010). These four adolescent problem gambling assessment tools range in number of items from seven items on the MAGS, 12 items on the DSM-IV-J and SOGS-RA, and 44 items on the CAGI, although nine of those 44 items are used to identify problem gambling. While seven items may be considered “brief” there are some settings and screening purposes where a smaller number of items is required, such as student surveys that attempt to screen for a large number of risky behaviors with the fewest number of items, such as the Minnesota Student Survey (Stinchfield, 2011). Therefore, there is a need for a brief screen for adolescent problem gambling.

How should a brief screen for adolescent problem gambling be developed? Stinchfield (2010) reported that most adolescent problem gambling measurement tools are scales originally developed for adults and then later adapted for adolescents, such as the SOGS-RA and DSM-IV-J. Because these scales were originally developed for adults, their content may not be applicable to adolescents, and therefore may not be appropriate as a source of items for adolescents. In contrast, the CAGI was developed from the outset specifically for adolescents (Wiebe et al., 2005, 2008). A consortium of Canadian provincial funding organizations directed a four-member research team to develop a measure of adolescent problem gambling under the supervision of the Canadian Centre on Substance Abuse (CCSA). The four-member research team included Dr. Jamie Wiebe of Ontario, Dr. Harold Wynne of Alberta, Dr. Joel Tremblay of Quebec, and Dr. Randy Stinchfield of Minnesota. Adolescents were included in the development of the CAGI and participated in focus groups to review and edit the content of the CAGI to make sure it was appropriate and relevant for adolescents. The timeframe of the CAGI inquires about gambling behaviors in the past 3 months to match an adolescent's focus on recent activities rather than the distant past, particularly since adolescence is a time of rapid changes and development. An adolescent's behavior of a year ago may not reflect their current behavior at all. The CAGI measures how often and how much time adolescents play 19 types of gambling; and two items inquire about the amount of money (or items of value) lost gambling. The CAGI purports to measure four gambling-related domains of loss of control, and social, psychological, and financial consequences. A fifth scale, Gambling Problem Severity Scale (GPSS), purports to identify problem gambling. It should be noted that many of the CAGI items did not originate with the CAGI, but rather were borrowed from other instruments and found to be reliable, valid, and accurate for the new CAGI scales. The CAGI was found to yield satisfactory estimates of reliability, validity and classification accuracy (Tremblay et al., 2010).

Because the CAGI was the only assessment tool developed specifically for adolescents and has items written by and for adolescents, and because it has demonstrated satisfactory psychometric properties, the CAGI was chosen as the source of items for the development of a new brief screen. Furthermore, the GPSS of the CAGI was developed from a large pool of diagnostic items that was narrowed down to a set of nine items that demonstrated classification accuracy for Gambling Disorder among adolescents. These nine items served as the item pool from which a brief screen for adolescent problem gambling was developed. The purpose of this study is to develop and evaluate the initial reliability, validity, and classification accuracy of a new brief screen for adolescent problem gambling.

## Method

### Participants

Data were used from 105 Canadian adolescents who were recruited for the CAGI development study in 2008 and 2009 from schools in Manitoba and Quebec (*n* = 66; males = 32; females = 34) and seven clinics in Quebec (*n* = 39; males = 24; females = 15). The sample ranged in age from 12 to 19 years; 84% were White; and 83% were from Quebec and spoke French and 17% were from Manitoba and spoke English. The goal of this recruitment from both school and clinical settings was to find a sufficient number of adolescents with gambling problems for the development of an adolescent problem gambling instrument. This study used existing data from the 2008 to 2009 CAGI development study with no individual identifiers and does not involve living human subjects and therefore is exempt from ethics review. The original CAGI development research from which this data was obtained had ethics approval: Ethical committee for Addiction Specialized Treatment Centers in Quebec, certificate number: CERT/2005-040. For more details about this sample, please see Tremblay et al. (2010) and Wiebe et al. (2008).

### Instruments

The CAGI is a 44-item paper-and-pencil questionnaire that can be administered in 20 min. The CAGI goes beyond a simple single scale to measure gambling by measuring multiple domains of gambling problem severity. The CAGI has 19 items that measure gambling frequency using six-point response options and time spent gambling in a typical week on 19 forms of gambling and two items to measure money and items of value lost gambling. One of the 19 forms of gambling is a fake game called “Blotzito” to measure invalid and inattentive responding, response distortion and exaggeration or faking bad. The second half of the CAGI measures five problem gambling-related domains: (a) Gambling Problem Severity Scale (GPSS; 9 items); (b) psychological consequences (6 items); (c) social consequences (5 items); (d) financial consequences (6 items); and (e) loss of control (4 items). The intent of the developers of the CAGI was to measure the continuum and the complexity of gambling behavior, rather than a dichotomy of either presence or absence of problem gambling as is found in most existing adolescent and adult instruments. The developers also wanted to produce an instrument that would be useful for epidemiological studies as well as for clinical and school settings. Early estimates of reliability, validity, and classification accuracy are satisfactory including reliability coefficient *alphas* ranging from 0.83 to 0.90, temporal stability coefficients ranging from 0.77 to 0.90; convergent validity coefficients ranging from *r* = 0.14 to 0.67; and for the GPSS *sensitivity* = 0.93 and *specificity* = 0.93 (Tremblay et al., 2010).

The reference standard against which the new brief screen was tested was DSM-5 diagnostic criteria for Gambling Disorder (GD). DSM-5 GD was measured with Stinchfield's DSM-IV diagnostic criteria for Pathological Gambling, a self-administered paper-and-pencil questionnaire (as well as clinician-administered interview) which is part of the Gambling Treatment Outcome Monitoring System (GAMTOMS; Stinchfield et al., 2007) and has been revised for DSM-5 (Stinchfield et al., 2016). These same 10 diagnostic items were included in a clinician-administered diagnostic interview to obtain a DSM-5 diagnosis of GD. The reference standard was a combination of the adolescent's self-report on a paper-and-pencil questionnaire to measure DSM-5 diagnostic criteria for GD and the clinician-administered diagnostic interview for GD. Both the adolescent and the clinician had to have GD present for the case to be in the GD group.

Stinchfield's measure of DSM-IV diagnostic criteria for Pathological Gambling includes 10 items, one item for each criterion, paraphrased from the 10 DSM-IV diagnostic criteria for Pathological Gambling (American Psychiatric Association, 1994). The DSM-IV measure was adapted to measure DSM-5 diagnostic criteria for Gambling Disorder (American Psychiatric Association, 2013) for this study by deleting the illegals acts criterion, resulting in nine criteria, and using a cut score of four to diagnose GD, rather than five as in DSM-IV. See Appendix A for a copy of Stinchfield's measure of DSM-IV diagnostic criteria of Pathological Gambling and rules for adaptation to DSM-5. This measure has demonstrated satisfactory reliability with internal consistency estimates of Cronbach's *alpha* ranging from 0.87 to 0.98 for a combined community and gambling treatment sample (Stinchfield et al., 2016), and temporal stability as measured by 1-week test-retest was *Intraclass Correlation* = 0.71 (Stinchfield et al., 2016). In terms of convergent validity, the DSM-5 GD scale was correlated with the SOGS *r* = 0.97 (Stinchfield et al., 2016). In terms of classification accuracy, using the DSM-5 cut score of four to indicate a diagnosis of GD (American Psychiatric Association, 2013), and using a reference standard of group membership (clinical vs. community), this scale yielded a *hit rate* range from 0.90 to 0.99, *sensitivity* range from 0.88 to 0.98, and *specificity* range from 0.83 to 0.99, all of which are satisfactory (Stinchfield et al., 2016).

The South Oaks Gambling Scale—Revised for Adolescents (SOGS-RA) is an adaptation of the South Oaks Gambling Scale (SOGS; Lesieur and Blume, 1987) for adolescents (Winters et al., 1993, 1995). The SOGS-RA purports to measure signs and symptoms of problem gambling and negative consequences over the past year. The SOGS-RA consists of 12 items with yes/no response options. A score of four or more indicates problem gambling. The SOG-RA has demonstrated satisfactory evidence of reliability, validity and classification accuracy (Stinchfield, 2010).

### Procedures

As stated earlier, this study relied on a secondary analysis of existing data from a sample of 105 adolescents who participated in the 2008–2009 CAGI development study (Tremblay et al., 2010). The sample was recruited from schools in Manitoba and Quebec; and seven clinics in Quebec. In Manitoba, participation required student and parental consent. In Quebec, parental consent was required for students 13 years of age or younger. Teachers read a consent form aloud in their classroom and the students were given a consent form to take home for their parents/guardians to read and sign. Students were informed that their answers would be kept confidential and that their names would not be used on the questionnaire. After signed consent forms were obtained, the CAGI was administered via paper-and-pencil questionnaire. Following in-class administration of the CAGI and upon student consent of follow-up contact, researchers invited 200 of the highest frequency gamblers to participate in a follow-up assessment that included administration of Stinchfield's measure of DSM-IV diagnostic criteria for Pathological Gambling and a clinical interview. In total, 109 students participated in the clinical interview, however, 43 interviews were not retained for analysis because the student reported gambling on a fictitious gambling activity named “Blotzito” (*n* = 5), missing data from follow-up assessment (*n* = 1), or too long a delay (>4 weeks) between class administration of CAGI and the clinical interview (*n* = 37). Sixty-six valid student interviews were retained.

The clinical setting included clinics for youth with problem behavior and substance abuse. New clients were screened during the admission process using the SOGS-RA. Clients scoring three or more were informed of the study and, if interested, signed a form giving a research team member authorization to contact them. Once the consent form was signed, the participant was administered the CAGI, Stinchfield's measure of DSM-IV diagnostic criteria for Pathological Gambling, and a clinical interview. The clinical interview included a copy of the DSM-IV diagnostic criteria for Pathological Gambling and interviewers were asked to endorse each criterion they judged to be present for each participant. Participants from the school settings were not administered the SOGS-RA. The interviewers were five clinicians, including four females and one male, two had a master's degree in psychology and two had a baccalaureate degree in social work and one was a doctoral student. All of them, except the doctoral student, were clinicians specialized in the treatment of problem gambling and working in a specialized addiction treatment center. Their years of experience ranged from 7 to 20 years, except for the doctoral student who had 1 year of experience from her clinical work as a doctoral student. The interviewers were trained in problem gambling assessment. They were hired to conduct these interviews. For more details of the procedures, please see Tremblay et al. (2010).

### Data analyses for screen development and psychometric evaluation

Screen development included three procedures. First, all nine GPSS items were entered into a stepwise multivariate Discriminant Function Analysis (DFA) with DSM-5 diagnosis of GD as the dependent variable. The goal was to identify the best items for classification of GD diagnosis.

Second, the smaller set of items identified by the DFA were summed and a cut score was determined by examining the frequency distributions of the new screen in the GD and No GD groups, and computing classification accuracy indices for different cut scores. Classification accuracy was assessed with standard accuracy indices including hit rate (diagnostic efficiency), sensitivity, and specificity (Fleiss, 1981; Baldessarini et al., 1983; Friedman and Cacciola, 1998) with cut score selection based on maximizing classification accuracy and balancing false positive and false negative errors. Since a brief screen would likely be used in various settings and for different purposes it was decided to balance false positive and false negative errors, rather than give preference to one type of diagnostic error over another in the cut score selection.

Third, psychometric evaluation included computations of reliability, validity and classification accuracy of the new screen (Nunnally, 1978; Allen and Yen, 1979; American Educational Research Association, American Psychological Association, National Council on Measurement in Education, 1999). Reliability was examined by computing both Cronbach's (1951) coefficient *alpha;* and McDonald's coefficient *omega* (McDonald, 1985; Gadermann et al., 2012) which is recommended for estimating reliability of items with ordinal response options. Cronbach's *alpha* and McDonald's *omega* are interpreted on a scale from 0 to 1. The higher the *alpha or omega*, the better the reliability of the scale. Coefficient *alpha* is effected by the number of items in a scale, the larger the number of items the higher the internal consistency, such that a coefficient *alpha* on a brief screen will be attenuated by the few number of items. As a criterion, Nunnally (1978) suggests that scales have an *alpha* of 0.70 or greater to be considered as having a minimal level of internal consistency for research purposes.

Validity was examined by measures of convergent validity. Convergent validity refers to how well a scale correlates or converges with an alternate measure of the same construct. Convergent validity was examined by measuring the relationship between the screen and the SOGS-RA. The SOGS-RA is a measure of problem gambling adapted for adolescents, and therefore the new screen should be related to the sum of the 12 SOGS-RA items. To demonstrate evidence of convergent validity, the screen should obtain moderate to high correlations (*r* > 0.30) with other measures of the same construct (Cichetti, 1994).

Classification accuracy was measured by computing standard diagnostic statistics of hit rate (diagnostic efficiency), sensitivity, specificity, false negative rate, and false positive rate (Fleiss, 1981; Baldessarini et al., 1983; Friedman and Cacciola, 1998). In order to demonstrate satisfactory classification accuracy, the hit rate (diagnostic efficiency), sensitivity and specificity must all be 0.80 or greater (Cichetti, 1994; Glascoe, 2005; DiStefano and Morgan, 2011).

To compute the DFA and the classification accuracy analyses a reference or “gold” standard is used against which to compare the test. There is no consensus among investigators about what to use for a reference standard for diagnosing GD, so investigators have used standardized diagnostic interviews or group membership (general population vs. a GD treatment sample). In this study, to create a reference standard, the DSM-5 diagnosis of GD was determined by the combination of the adolescent's self-report on a paper-and-pencil questionnaire to measure DSM-5 diagnostic criteria for GD and the clinician administered diagnostic interview of DSM-5 diagnostic criteria for GD. Both the adolescent and the clinician had to have GD present for the case to be in the GD group. The inter-rater agreement between adolescent self-administered questionnaire and clinician-administered diagnostic interview for DSM-5 diagnosis of GD was *kappa* = 0.76, which is excellent agreement (Cohen, 1960; Fleiss, 1981). A kappa value > 0.75 generally indicates “excellent” agreement, a value between 0.40 and 0.75 indicates “satisfactory” agreement, and a value <0.40 indicates “poor” agreement (Fleiss et al., 2003).

## Results

### Selection of items for the brief adolescent gambling screen (BAGS)

The nine items of the CAGI GPSS were entered into a stepwise multivariate DFA with DSM-5 diagnosis of GD as the dependent variable. The stepwise multivariate DFA yielded three items as the best predictors of membership in the GD group and maximized classification accuracy. Table 1 shows the best or strongest predictor GPSS items selected from the DFA along with the unstandardized canonical discriminant function coefficient for each item. Items are ordered by magnitude of the unstandardized canonical discriminant function coefficient which is the weight of the item in an equation to classify each adolescent into the GD or No GD group. Item weights, along with a constant, are used in an equation to compute a score for each case. The score for each case is then compared to the group centroid for each of the two groups and whichever centroid the case score is closest to, is the group assignment for that case.

**Table 1:** Brief Adolescent Gambling Screen (BAGS): Three best CAGI GPSS items and Unstandardized Canonical Discriminant Function Coefficients (UCDFC).

**BAGS item (CAGI gambling problem severity scale item)**	**UCDFC**
BAGS #1 (CAGI #26). Skipped hanging out with friends who do not gamble/bet	1.265
BAGS #2 (CAGI #40). Felt that you might have a problem with gambling/betting	0.868
BAGS #3 (CAGI #37). Hidden your gambling/betting from your parents, other family members or teachers	0.483
Discriminant Function equation = (Constant = −1.296) + (CAGI #26 ^*^ 1.265) + (CAGI #40 ^*^ 0.868) + (CAGI #37 ^*^ 0.483). Group Centroid for No Gambling Disorder = −0.834; Group Centroid for Gambling Disorder = 2.814	

*Items are rank ordered by magnitude of the Unstandardized Canonical Discriminant Function Coefficient (UCDFC)*.

### Cut score selection and classification accuracy of the BAGS

The BAGS could be scored using the DFA equation including the item weights, however, this adds a layer of complication for screen users and it is likely not much more accurate than using a summed raw score derived by summing the response option from each item (0, 1, 2, or 3). Furthermore, item weights can vary by population and therefore the item weights from this sample may be unique and may not generalize to a different sample.

The BAGS has three items with four-point response options that are coded as 0–3, for a total score range of 0–9. Table 2 shows a cross-tabulation of the frequency distribution of BAGS scores from 0 to 9 for the two groups by Gambling Disorder and No Gambling Disorder, along with the probability. There was an increasing probability of having a GD with increasing BAGS score. A score of 0, 1, 2, or 3 resulted in almost no chance of having a GD. A score of four or greater indicated a very high likelihood of GD, and scores of 6 or greater indicated certainty of having GD. Therefore, a cut score of four maximized classification accuracy and balanced false positive and false negative classification errors. A cross-tabulation of the BAGS cut score of four and GD is shown in Table 3. The BAGS yielded satisfactory evidence of classification accuracy with hit rate, sensitivity and specificity of 0.95, 0.88, and 0.98, respectively. It should be noted that the same sample of adolescents was used to select the items and compute classification accuracy and this likely inflates classification accuracy.

**Table 2:** Probability of DSM-5 Gambling Disorder (GD) for each BAGS Score from 0 to 9.

**BAGS score**	**DSM-5 GD Status**		**Probability of GD**
	**GD**	**No GD**	
0	0	57	0/57 = 0%
1	0	7	0/7 = 0%
2	2	7	2/9 = 22%
3	1	8	1/9 = 11%
4	5	1	5/6 = 83%
5	7	1	7/8 = 88%
6	4	0	4/4 = 100%
7	4	0	4/4 = 100%
8	1	0	1/1 = 100%
9	0	0	0/0 = 0%

**Table 3:** Crosstabulation of the BAGS and DSM-5 Gambling Disorder.

**BAGS cut score**	**DSM-5 GD**		**Row totals**
	**Gambling disorder**	**No gambling disorder**	
4+	21	2	23
<4	3	79	82
Column Totals	24	81	105
Base Rate = 24/105 = 0.23			
Hit Rate = (21 + 79)/105 = 0.95			
Sensitivity = 21/24 = 0.88			
Specificity = 79/81 = 0.98			
False Positive Rate = 2/81 = 0.02			
False Negative Rate = 3/24 = 0.12			

For purposes of comparison, the cross-tabulation for the nine-item CAGI GPSS and GD is shown in Table 4, along with classification accuracy indices. The CAGI GPSS yielded satisfactory classification accuracy with hit rate, sensitivity and specificity of 0.89, 1.00, and 0.85, respectively. The BAGS had a higher hit rate and specificity, but lower sensitivity than the CAGI GPSS. The CAGI GPSS had no false negative cases, but 12 false positive cases due to its design to minimize false negative errors, the more serious classification error in clinical settings. The BAGS balanced classification errors with three false negative errors and two false positive errors.

**Table 4:** Crosstabulation of the CAGI GPSS and DSM-5 Gambling Disorder.

**CAGI GPSS cut score**	**DSM-5 GD**		**Row totals**
	**Gambling disorder**	**No gambling disorder**	
6+	24	12	36
<6	0	69	69
Column Totals	24	81	105
Base Rate = 24/105 = 0.23			
Hit Rate = (24 + 69)/105 = 0.89			
Sensitivity = 24/24 = 1.00			
Specificity = 69/81 = 0.85			
False Positive Rate = 12/81 = 0.15			
False Negative Rate = 0/24 = 0.00			

For purposes of comparison, the cross-tabulation for the SOGS-RA (using a standard cut score of four) and GD for the 39 adolescents who had both the SOGS-RA and GD, is shown in Table 5 and the cross-tabulation of the BAGS for the same 39 adolescents is shown in Table 6. The SOGS-RA did not yield satisfactory classification accuracy with hit rate, sensitivity and specificity of 0.64, 0.87, and 0.31, respectively. Only sensitivity was above the minimum criterion of 0.80 for satisfactory classification. The BAGS had a higher hit rate, sensitivity and specificity, than the SOGS-RA and all of the BAGS classification accuracy coefficients were above the minimum criterion of 0.80.

**Table 5:** Crosstabulation of the SOGS-RA and DSM-5 Gambling Disorder in subsample with SOGS-RA (*n* = 39).

**SOGS-RA cut score**	**DSM-5 GD**		**Row totals**
	**Gambling disorder**	**No gambling disorder**	
4+	20	11	31
<4	3	5	8
Column Totals	23	16	39
Base Rate = 23/39 = 0.59			
Hit Rate = (20 + 5)/39 = 0.64			
Sensitivity = 20/23 = 0.87			
Specificity = 5/16 = 0.31			
False Positive Rate = 11/16 = 0.69			
False Negative Rate = 3/23 = 0.13			

**Table 6:** Crosstabulation of the BAGS and DSM-5 Gambling Disorder in subsample with SOGS-RA (*n* = 39).

**BAGS cut score**	**DSM-5 GD**		**Row totals**
	**Gambling disorder**	**No gambling disorder**	
4+	21	1	22
<4	2	15	17
Column Totals	23	16	39
Base Rate = 23/39 = 0.59			
Hit Rate = (21 + 15)/39 = 0.92			
Sensitivity = 21/23 = 0.91			
Specificity = 15/16 = 0.94			
False Positive Rate = 1/16 = 0.06			
False Negative Rate = 2/23 = 0.09			

### Reliability

Reliability of the BAGS as measured by Cronbach's (1951) coefficient *alpha* was 0.72; and as measured by McDonald's coefficient *omega* was 0.79.

### Validity

Convergent validity coefficient for BAGS and SOGS-RA, *r* = 0.67.

## Discussion

The purpose of this study was to develop and evaluate the psychometric properties of a new brief screen to measure problem gambling among adolescents. This new brief screen was developed from the CAGI, an assessment tool that was specifically designed for adolescents, and therefore this is an advantage over using questions developed for adults and then later adapted for adolescents, as was done with the SOGS-RA. The three items were identified from a statistical procedure, stepwise multivariate discriminant function analysis, that is used to select the best items from a pool of items in order to accurately classify cases into two groups. The statistical procedure selected three of the nine CAGI GPSS items that were the best predictors of group membership (GD vs. No GD). These three items make up the new Brief Adolescent Gambling Screen (BAGS) and this new screen is in the public domain, that is, free of charge to use.

Next, the psychometric properties, reliability, validity, and classification accuracy, of the new screen were measured and compared to *a priori* criterion levels for each property and psychometric standards for behavioral instruments (Nunnally, 1978; Allen and Yen, 1979; American Educational Research Association, American Psychological Association, National Council on Measurement in Education, 1999). The reliability of the BAGS was measured with Cronbach's *alpha* and was 0.72, which is just above the minimum level of reliability, *alpha* > 0.70, for research purposes (Nunnally, 1978). The number of items in a scale effects the magnitude of Cronbach's *alpha* such that fewer items attenuate *alpha* and this must be considered in the context of a three-item brief screen. The reliability of the BAGS as measured by McDonald's *omega* was 0.79 and this initial estimate of reliability is satisfactory.

The convergent validity of the BAGS was measured by correlation with the adolescent self-administered SOGS-RA (*r* = 0.67). This validity coefficient was above the minimum of *r* > 0.30 (Cichetti, 1994) and shows preliminary evidence for the validity of the BAGS.

The BAGS has a score range of 0–9 and a cut score of four maximized classification accuracy and balanced false positive and false negative errors. The classification accuracy of the BAGS was measured by computing standard diagnostic statistics of hit rate (diagnostic efficiency), sensitivity, specificity, false negative rate, and false positive rate (Fleiss, 1981; Baldessarini et al., 1983; Friedman and Cacciola, 1998). The BAGS yielded satisfactory evidence of classification accuracy with hit rate, sensitivity, and specificity of 0.95, 0.88, and 0.98, respectively, all of which are above the minimum criterion for satisfactory classification accuracy of 0.80 (Cichetti, 1994; Glascoe, 2005; DiStefano and Morgan, 2011). It should be noted that this sample of adolescents was used to select the BAGS items and this procedure likely inflates classification accuracy.

For comparison purposes, the accuracy of the BAGS was compared to that of the CAGI GPSS and SOGS-RA. The BAGS had a higher hit rate and specificity, but lower sensitivity than the CAGI GPSS. The BAGS had higher hit rate, sensitivity, and specificity than the SOGS-RA. The BAGS was equivalent to the CAGI GPSS and more accurate than the SOGS-RA, however, it should be noted that both the CAGI GPSS and the SOGS-RA have cut scores that are designed to minimize false negative errors at the expense of more false positive errors, whereas the BAGS cut score was designed to balance false negative and false positive errors and this likely explains differences in the classification accuracy of the BAGS compared to the CAGI GPSS and SOGS-RA. If the BAGS is used in anonymous adolescent surveys, the cut score of 4+ can be used to obtain a prevalence estimate. However, if the BAGS is to be used to identify adolescents for further assessment and diagnosis, then the cut score may need to be lowered in order to minimize false negative errors. Based on the sample used in this study, a cut score of 2+ would eliminate false negative errors (0/24 = 0), however this lower cut score would also inflate false positive errors (17/81 = 0.21) and that is the tradeoff for no false negative errors.

A note about the source of these three items. Two of these three items, while borrowed from the CAGI for this study, do not originate from the CAGI, but rather were adapted for the CAGI from other sources. The item, “How often have you felt that you might have a problem with gambling/betting?” can be traced to the SOGS (Lesieur and Blume, 1987) and it is also included in the SOGS-RA (Winters et al., 1993) and Canadian Problem Gambling Index (CPGI; Ferris and Wynne, 2001). The item, “How often have you hidden your gambling/betting from your parents, other family members or teachers?” can be found in the SOGS, SOGS-RA, DSM-IV, and DSM-5 diagnostic criteria (American Psychiatric Association, 2013). The item “How often have you skipped hanging out with friends who do not gamble/bet to hang out with friends who do gamble/bet?” was written by the CAGI development team and was inspired by adolescent substance abuse instruments.

### Limitations and future research directions

There are limitations of this study that need to be noted. First, the data are based on adolescent self-report and there is no objective verification of the accuracy of this information. However, efforts were made to enhance the validity of self-report by informing respondents that their answers would be kept confidential and participants were informed that their names would not be used on instruments. Nevertheless, the data are dependent on self-report and further research needs to be conducted on the validity of self-report about gambling behaviors. Second, classification accuracy was computed from the sample used to compute the discriminant function and this maximizes classification accuracy. Therefore, these results need to be cross-validated on other samples and in different settings. Third, the results are based on a relatively small sample of adolescents. Therefore, the BAGS should be cross-validated on larger and more diverse samples of adolescents, including non-white adolescents.

In summary, the BAGS demonstrated satisfactory reliability, validity, and classification accuracy and in this preliminary study, the BAGS yielded equivalent accuracy to the CAGI GPSS and better accuracy than the SOGS-RA. The BAGS can be used in those projects limited to a small number of items to screen for adolescent problem gambling. Different cut scores are recommended for different purposes. For anonymous surveys where the goal is a sample or population prevalence rate, a cut score of four or more is recommended to balance false negative and false positive errors. For clinical settings or for purposes of identifying individuals who require further assessment and a diagnostic interview, a cut score of two or more is recommended to minimize false negative errors (which will raise the false positive rate). A sign of a maturing scientific field is that the instruments used to measure the phenomenon of interest become more precise, and it is the intent of this study to improve the screening and assessment of adolescent problem gambling.

## Author contributions

RS was the lead author and was involved in all aspects of the literature review, statistical analyses, and reporting of the results. HW, JW, and JT were involved in all aspects of the project and writing and each had an area or two of focus. HW focused on the conceptualization of adolescent problem gambling, JW and JT focused on project management and JT also focused on the statistical analyses. All four authors were involved in the writing and editing of the manuscript.

### Conflict of interest statement

The authors declare that the research was conducted in the absence of any commercial or financial relationships that could be construed as a potential conflict of interest.

## Appendix a

Stinchfield's measure of DSM-IV diagnostic criteria for Pathological Gambling.

For this adolescent study, a time period of “During the past 3 months” was used to match the CAGI time period.

**Table:**

1. Have there been periods when you spent a lot of time thinking about past gambling experiences, thinking about future gambling ventures, or thinking about ways of getting money with which to gamble?	Yes	No
2. Have you needed to gamble with larger amounts of money or with larger bets in order to obtain the same feeling of excitement?	Yes	No
3. Have you tried to cut down or stop your gambling several times in the past and been unsuccessful?	Yes	No
4. Did you feel quite restless or irritable after you tried to cut down or stop gambling?	Yes	No
5. Do you feel that you gamble as a way to run away from personal problems or to relieve uncomfortable emotions, such as nervousness or sadness?	Yes	No
6. After you lose money gambling, do you often return another day to try to win back your losses?	Yes	No
7. Have you lied to family members, friends, or others in order to hide your gambling from them?	Yes	No
8. Have you committed any illegal acts (such as theft, forgery, embezzlement, or fraud) to finance your gambling?	Yes	No
9. Have you almost lost or actually lost a relationship with someone important to you, or a job, or school or career opportunity because of gambling?	Yes	No
10. Have you relied on others to bail you out and pay your gambling debts or to pay your bills when you have financial problems caused by gambling?	Yes	No

Scoring Instructions: For DSM-IV, five or more items endorsed with a “Yes” answer, indicate Pathological Gambling. To adapt for DSM-5: exclude criterion #8; and use cut score of four or more items endorsed with a “Yes” answer, to indicate Gambling Disorder.
